# HUWE1 regulates mitophagy to protect dopaminergic neurons from 6-OHDA- and MPP⁺-induced neurotoxicity

**DOI:** 10.1007/s10565-026-10146-7

**Published:** 2026-02-05

**Authors:** Chanhaeng Lee, Dong Yeol Kim, Sang-Min Kim, Inn-Oc Han

**Affiliations:** 1 Department of Physiology and Biophysics, College of Medicine, Inha University, 100 Inha Ro, Michuhol-Gu, Incheon, 22212 Korea; 2 Department of Biomedical Science, Program in Biomedical Science and Engineering, Inha University, Incheon, Korea

**Keywords:** HUWE1, Mitophagy, Parkinson’s disease, SH-SY5Y cells, Dopaminergic neurons

## Abstract

Parkinson’s disease (PD) is characterized by dopaminergic neuronal loss, often associated with mitochondrial dysfunction and impaired mitophagy. Here, we investigated the role of HUWE1, an E3 ubiquitin ligase, in regulating mitophagy and neuronal survival in a cellular PD model. HUWE1 promoted mitophagy, whereas its depletion sensitized SH-SY5Y cells to 6-hydroxydopamine (6-OHDA)- and 1-methyl-4-phenylpyridinium (MPP⁺)-induced cytotoxicity and mitochondrial dysfunction. Notably, both toxins downregulated HUWE1, suggesting that loss of HUWE1 contributes to dopaminergic vulnerability. Conversely, HUWE1 overexpression preserved mitochondrial integrity and enhanced mitophagy under neurotoxic stress. Importantly, BL-918, a ULK1 activator that promotes AMBRA1 recruitment, facilitated HUWE1-mediated mitophagy in SH-SY5Y cells. BL-918 treatment significantly attenuated 6-OHDA- and MPP⁺-induced neurotoxicity and protected mitochondrial function via HUWE1 activation. Collectively, these findings identify HUWE1 as a key mechanistic regulator of mitophagy linked to dopaminergic neuronal vulnerability, and provide a conceptual framework for future investigations examining its role in PD–relevant model systems.

**Graphical Abstract:**

Highlights

• HUWE1 is an E3 ubiquitin ligase that regulates mitophagy in dopaminergic neurons.

• Neurotoxicity induced by 6-OHDA or MPP^+^ reduces HUWE1 expression, thereby inhibiting mitophagy in dopaminergic neurons.

• Overexpression or activation of HUWE1 promotes the clearance of damaged mitochondria under neurotoxic stress.

• BL-918 enhances HUWE1-mediated mitophagy and mitigates neurotoxin-induced mitochondrial dysfunction in dopaminergic neurons.

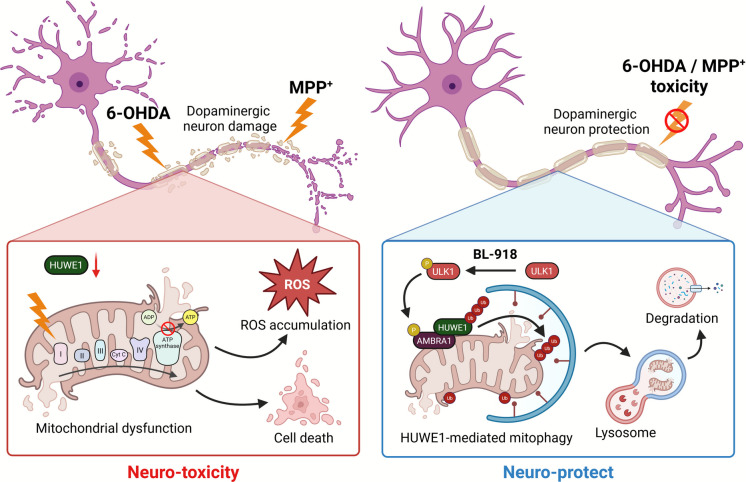

## Introduction

Parkinson’s disease (PD) is a progressive neurodegenerative disorder characterized by the selective loss of dopaminergic neurons in the substantia nigra pars compacta (SNpc), leading to reduced dopamine levels in the striatum (ST) and the development of cardinal motor symptoms such as bradykinesia, rigidity, and resting tremor (Kalia & Lang 2015; Poewe et al. 2017). The pathogenesis of PD is multifactorial, involving impaired protein homeostasis (Sharma et al. 2025), dysfunction of proteasomal and lysosomal degradation pathways (Dai et al. 2024), disturbances in protein and membrane trafficking (Karami et al. 2025), synaptic dysfunction (Rosh et al. 2024), neuroinflammation (Lind-Holm Mogensen et al. 2025), and mitochondrial impairment (Tingting et al. 2025). Among these, mitochondrial dysfunction is increasingly recognized as a central pathogenic event, as impaired oxidative phosphorylation, elevated reactive oxygen species (ROS), and bioenergetic failure critically compromise dopaminergic neuronal survival (Tingting et al. 2025). Thus, therapeutic strategies aimed at restoring mitochondrial quality control are considered promising therapeutic approaches in PD.

Experimental modeling of dopaminergic neurodegeneration frequently employs neurotoxins such as 6-hydroxydopamine (6-OHDA) and 1-methyl-4-phenylpyridinium (MPP⁺), which selectively impair dopaminergic neurons by inhibiting mitochondrial complex I, increasing ROS production, and activating intrinsic apoptotic pathways (Lucchesi et al. 2025; Álvarez-Luquín et al. 2025). These models recapitulate key aspects of mitochondrial dysfunction observed in PD and provide valuable platforms for investigating mitochondrial quality control mechanisms.

Mitophagy, the selective autophagic removal of damaged or dysfunctional mitochondria, is a fundamental cellular process that preserves mitochondrial integrity and supports neuronal viability (Onishi et al. 2021). Under basal conditions, mitochondria undergo continuous turnover through mitophagy to maintain homeostasis, whereas stress-induced mitophagy is critical for preventing the accumulation of ROS and damaged organelles (Garza-Lombó et al. 2020; Xiao et al. 2022; Kelly et al. 2024). Canonically, the PINK1/Parkin pathway mediates mitophagy: mitochondrial depolarization stabilizes PINK1 on the outer mitochondrial membrane, recruiting Parkin to ubiquitinate mitochondrial proteins, thereby targeting them for autophagic degradation (Narendra et al. 2008; Pickrell and Youle 2015). Beyond this canonical pathway, evidence supports alternative ubiquitin-dependent mitophagy mechanisms that operate independently of PINK1/Parkin. One such alternative involves HUWE1 (HECT, UBA, and WWE domain-containing E3 ubiquitin ligase 1), which cooperates with activating molecule in BECN1-regulated autophagy protein 1 (AMBRA1) to mediate mitophagy. Upon mitochondrial stress, AMBRA1 translocates to the outer mitochondrial membrane, where it recruits HUWE1 to ubiquitinate mitochondrial substrates, promoting their autophagic degradation (Di Rita et al. 2018a; Strappazzon et al. 2015). ULK1 kinase further regulates this pathway by phosphorylating AMBRA1, enhancing its mitochondrial targeting and facilitating efficient HUWE1-mediated mitophagy (Li et al. 2022; Liu et al. 2023). Although the ULK1–AMBRA1–HUWE1 axis has been characterized in non-neuronal systems, its role in neurons, particularly dopaminergic neurons vulnerable to oxidative stress, remains largely unexplored.

In this study, we aimed to elucidate the role of HUWE1 in mitophagy and neuronal survival under PD-relevant mitochondrial stress. Using SH-SY5Y neuroblastoma cells exposed to 6-OHDA and MPP⁺, we investigated whether HUWE1 facilitates mitophagy to preserve mitochondrial integrity and enhance cell viability. Our findings delineate a previously unrecognized role of HUWE1 in the regulation of neuronal mitophagy and establish a mechanistic basis for future investigations of HUWE1-associated pathways in PD–relevant experimental models.

## Materials and methods

### Cell culture

Human SH-SY5Y neuroblastoma cells were obtained from the Korean Cell Line Bank (Seoul, Korea). Cells were maintained in Dulbecco’s modified Eagle’s medium (DMEM; HyClone, UT, USA) supplemented with 10% fetal bovine serum (FBS; HyClone) and 100 U/mL penicillin–streptomycin (HyClone). Cultures were grown at 37 °C in a humidified atmosphere containing 5% CO₂. To ensure experimental reliability, cell lines were routinely authenticated and screened for mycoplasma contamination before use.

### HUWE1 overexpression and knockdown in SH-SY5Y cells

For overexpression experiments, SH-SY5Y cells were transfected with Myc-tagged HUWE1 wild-type (pCMV6-Myc-HUWE1 WT) or a C4341S mutant (pCMV6-Myc-HUWE1 C4341S), previously described by Lee et al. (Lee et al. 2024), using jetOPTIMUS® DNA transfection reagent (Polyplus-transfection, Illkirch, France) according to the manufacturer’s instructions. Cells were harvested 48 h post-transfection for protein analysis.

For gene knockdown, cells were transfected with synthetic *HUWE1* siRNA (Genolution, Seoul, Korea) using Lipofectamine™ 2000 (Invitrogen, Carlsbad, USA) following the manufacturer’s protocol. Cells were collected 72 h after siRNA transfection for subsequent protein extraction. The *HUWE1* siRNA sequence was identical to that previously reported (Lee et al. 2024).

### Cell viability and cytotoxicity assays

Cell viability and cytotoxicity were assessed using the Cell Counting Kit-8 (CCK-8; Dojindo, Kumamoto, Japan) and the LDH Cytotoxicity Detection Kit (Dojindo), respectively. SH-SY5Y cells were transfected with plasmids (24 h) or siRNA (48 h) and seeded at a density of 1 × 10^4^ cells per well in 96-well plates. Cells were then exposed to 50 μM 6-hydroxydopamine (6-OHDA; Sigma-Aldrich, MO, USA) or 1 mM MPP⁺ (MedChemExpress, NJ, USA) in the presence or absence of 20 μM BL-918 (MedChemExpress) for 24 h. For the CCK-8 assay, reagent was added directly to the culture medium and incubated for 30 min at 37 °C, after which absorbance was measured at 450 nm using a microplate reader. For the LDH assay, culture supernatants were collected, and LDH activity was quantified following the manufacturer’s instructions, with absorbance recorded at 490 nm. Results were normalized to untreated controls.

### Western blot analysis

Total cellular proteins were extracted using RIPA lysis buffer supplemented with protease inhibitors and lysed on ice for 30 min. Lysates were centrifuged at 13,000 rpm for 30 min at 4 °C, and the resulting supernatants were collected for protein quantification using the Bradford assay (Bio-Rad, CA, USA). Equal amounts of denatured proteins were separated by SDS-PAGE and transferred onto nitrocellulose membranes. The membranes were blocked with 5% non-fat milk for 1 h at room temperature and incubated overnight at 4 °C with the following primary antibodies: anti-HUWE1 (1:1,000, #9482, Cell Signaling Technology, MA, USA), anti-TOMM20 (1:1,000, #42,406, CST), anti-β-actin (1:1,000, sc-47778, Santa Cruz, CA, USA), anti-GAPDH (1:1,000, sc-32233, Santa Cruz), anti-Myc (1:1,000, #9B11, CST), anti-COX IV (1:1,000, #3E11, CST), anti-LC3B (1:1,000, #2775, CST), anti-Bcl-2 (1:1,000, sc-783, Santa Cruz), anti-Bax (1:1,000, sc-6236, Santa Cruz), anti-Cleaved caspase-3 (1:1,000, #9664, CST), anti-DRP1 (1:1,000, #8570, CST), anti-MFN2 (1:1,000, #9482, CST), anti-OPA1 (1:1,000, #80,471, CST), anti-p-ULK1 (1:1,000, #5869, CST), anti-ULK1 (1:1,000, A8529, ABclonal, MA, USA), anti-AMBRA1 (1:1,000, A1083, ABclonal), and anti-Ubiquitin (1:1,000, #39269, CST). After washing, the membranes were incubated with HRP-conjugated secondary antibodies (anti-mouse or anti-rabbit, 1:5,000, Santa Cruz) for 1 h at room temperature. Protein bands were visualized using an enhanced chemiluminescence (ECL) detection system (Biomax, Kyunggi-Do, Korea), and band intensities were quantified using ImageJ software.

### Mitochondrial staining and immunofluorescence

SH-SY5Y cells were cultured on glass coverslips and stained with MitoTracker™ Red (Thermo Fisher Scientific, MA, USA) for 20 min at room temperature to label mitochondria. Cells were then fixed with 4% paraformaldehyde for 30 min, permeabilized with 0.1% Triton X-100 for 10 min, and blocked with 2% bovine serum albumin (BSA) in PBS. Cells were incubated overnight at 4 °C with primary antibodies against HUWE1 (1:1,000, #9482, Cell Signaling Technology, MA, USA), Myc (1:1,000, #9B11, CST), LC3B (1:1,000, #2775, CST), and AMBRA1 (1:1,000, A1083, ABclonal, MA, USA). After three washes with blocking buffer, cells were incubated for 1 h at room temperature with the corresponding Alexa Fluor-conjugated secondary antibodies (Alexa Fluor 488, 594, or 647). Nuclei were counterstained with DAPI, and fluorescence images were acquired using a confocal laser scanning microscope equipped with Airyscan (LSM980, Carl Zeiss, Oberkochen, Germany).

### RNA extraction and qRT-PCR

Total RNA was extracted from SH-SY5Y cells using TRIzol™ reagent (Invitrogen, CA, USA) according to the manufacturer’s instructions. Complementary DNA (cDNA) was synthesized from 2 μg of total RNA using GoScript™ Reverse Transcriptase (Promega, WI, USA). Quantitative real-time PCR (qRT-PCR) was performed using SYBR Green Real-Time PCR Master Mix (BIOFACT, Daejeon, Korea) on specific primers for each target gene. Primer sequences were as follows: HUWE1: F, GAGGAGTTGAAGCTACCTTGTG; R, CTGGTGTGATCCACTTTGG, SIRT1: F, TAGACACGCTGGAACAGGTTGC; R, CTCCTCGTACAGCTTCACAGTC, PPARGC1A: F, CCAAAGGATGCGCTCTCGTTCA; R, CGGTGTCTGTAGTGGCTTGACT, NFE2L2: F, CACATCCAGTCAGAAACCAGTGG; R, GGAATGTCTGCGCCAAAAGCTG, TFAM: F, GTGGTTTTCATCTGTCTTGGCAAG; R, TTCCCTCCAACGCTGGGCAATT, GAPDH: F, TCATCAGCAATGCCTCCTGC; R, GGCATGGACTGTGGTCATGA. Gene expression levels were normalized to GAPDH, and relative expression was calculated using the ΔΔCt method.

### Mitochondrial fraction isolation

Mitochondrial fractions were isolated using a modified subcellular fractionation protocol. SH-SY5Y cells were washed with PBS and resuspended in mitochondrial fractionation buffer containing 10 mM Tris–HCl (pH 7.4), 250 mM sucrose, 20 mM HEPES (pH 7.4), 5 mM MgCl₂, 10 mM KCl, 1 mM EDTA, 1 mM EGTA, 0.02% digitonin, and protease inhibitors. Cells were homogenized by repeated aspiration through a 26-gauge needle attached to a syringe (10–20 passes). Homogenates were centrifuged at 900 × g for 2 min at 4 °C to remove nuclei and unbroken cells. The resulting supernatant was further centrifuged at 10,000 × g for 20 min at 4 °C, and the pellet was collected as the mitochondrial fraction.

### Measurement of intracellular ROS

Intracellular reactive oxygen species (ROS) levels were measured using 2′,7′-dichlorofluorescin diacetate (DCFDA; Sigma-Aldrich, MO, USA). SH-SY5Y cells were seeded on glass coverslips and treated under experimental conditions. After treatment, cells were incubated with 5 μM DCFDA in serum-free medium for 20 min at 37 °C in the dark. Excess dye was removed by washing twice with PBS, and fluorescence images were acquired using a confocal laser scanning microscope equipped with Airyscan (LSM980, Carl Zeiss, Oberkochen, Germany) with excitation at 488 nm and emission collected at 525 nm. Fluorescence intensity was quantified using ImageJ software.

### Mitochondrial functional analysis

Mitochondrial membrane potential (ΔΨm) and mitochondrial reactive oxygen species (MitoROS) were assessed using JC-1 (MedChemExpress, NJ, USA) and MitoSOX Red (MedChemExpress), respectively. SH-SY5Y cells were seeded on glass coverslips and treated under experimental conditions. For ΔΨm measurement, cells were incubated with 5 μM JC-1 in culture medium for 20 min at 37 °C in the dark, washed twice with PBS, and immediately imaged using a confocal microscope equipped with Airyscan (LSM980, Carl Zeiss, Oberkochen, Germany). JC-1 aggregates (red fluorescence, high ΔΨm) and monomers (green fluorescence, low ΔΨm) were detected at excitation/emission wavelengths of 561/590 nm and 488/530 nm, respectively. Mitochondrial membrane potential was quantified as the red-to-green fluorescence ratio using ImageJ software.

For MitoROS measurement, cells were incubated with 5 μM MitoSOX Red in serum-free medium for 20 min at 37 °C in the dark, washed twice with PBS, and fluorescence images were acquired using the same confocal microscope at excitation/emission of 510/580 nm. Fluorescence intensity was quantified using ImageJ software.

### Measurement of cellular ATP levels

Cellular ATP content was determined using the ATP Determination Kit (Invitrogen, CA, USA) following the manufacturer’s instructions. SH-SY5Y cells were washed with PBS and lysed by boiling in sterile distilled water for 10 min. Lysates were centrifuged at 12,000 × g for 20 min at 4 °C to remove cellular debris, and the resulting supernatants were collected. ATP levels were quantified using a luminometer, and values were normalized to protein content or cell number as appropriate.

### Mitochondrial electron transport chain enzyme activity assay

Activities of mitochondrial Complex I and Complex IV were measured using the Complex I Enzyme Activity Microplate Assay Kit (ab109721, Abcam, Cambridge, UK) and the Complex IV Human Enzyme Activity Microplate Assay Kit (ab109910, Abcam), respectively, according to the manufacturer’s instructions. Briefly, protein samples were captured by complex-specific antibodies pre-coated on microplate wells. Complex I activity was determined by spectrophotometric monitoring of NADH oxidation at 450 nm using 300 μg of protein per sample, while Complex IV activity was assessed by monitoring cytochrome c oxidation at 550 nm using 100 μg of protein per sample. Enzyme activities were calculated from the change in absorbance over time using the extinction coefficient specific to each assay.

### Statistical analysis

All experiments were independently performed at least three times using biological replicates. Data are presented as mean ± standard error of the mean (SEM). Statistical comparisons between two groups were conducted using an unpaired Student’s *t*-test, with p-values less than 0.05 considered statistically significant. For comparisons involving two experimental groups, statistical significance was conducted using an unpaired Student’s t-test. For comparisons involving three or more experimental groups, one-way ANOVA followed by Tukey’s post hoc test was used, as the experiments were designed to compare distinct treatment conditions within a neurotoxin-induced stress paradigm rather than a fully crossed factorial design. All analyses were performed using GraphPad Prism software (version 6.00; GraphPad Software, CA, USA).

## Results

### HUWE1 promotes mitophagy in SH-SY5Y cells

**Fig. 1:**
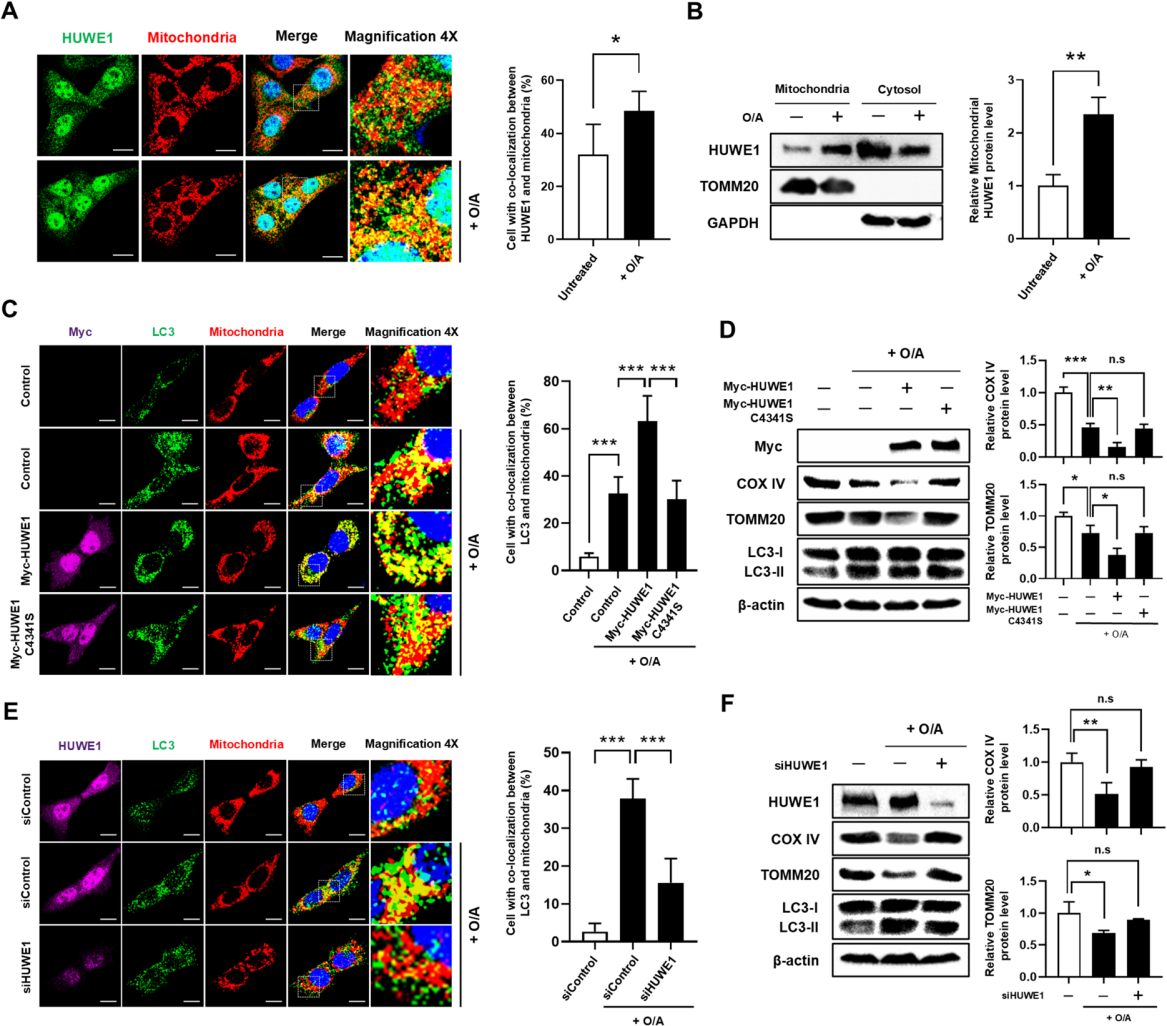
HUWE1 promotes mitophagy in SH-SY5Y cells. SH-SY5Y cells were subjected to mitochondrial stress by treatment with oligomycin and antimycin A (O/A) for 8 h. (**A**) Immunofluorescence analysis showing co-localization of endogenous HUWE1 (green) with mitochondria (red) in O/A-treated cells. Nuclei were stained with DAPI (blue). Scale bar, 10 μm. (**B**) Western blot analysis of HUWE1 protein levels in mitochondrial fractions of SH-SY5Y cells after O/A treatment. (**C**) Immunofluorescence analysis of LC3 (green) and mitochondria (red) in SH-SY5Y cells transfected with Myc-HUWE1 or catalytically inactive Myc-HUWE1^C4341S and treated with O/A. Scale bar, 10 μm. (**D**) Western blot analysis of Myc, LC3, COX IV, and TOMM20 in SH-SY5Y cells expressing Myc-HUWE1 or Myc-HUWE1^C4341S under O/A treatment. (**E**) Immunofluorescence analysis of LC3–mitochondria co-localization in SH-SY5Y cells transfected with *HUWE1* siRNA and treated with O/A. Scale bar, 10 μm. (**F**) Western blot analysis of HUWE1, LC3, COX IV, and TOMM20 in HUWE1-depleted SH-SY5Y cells under O/A treatment. Data are presented as mean ± SEM from at least three independent experiments. Panels A and B were analyzed using an unpaired two-tailed Student’s t-test, whereas panels C–F were analyzed using one-way ANOVA followed by Tukey’s post hoc test. Statistical significance: *P < 0.05, **P < 0.01, ***P < 0.001; n.s., not significant (*P* > 0.05)

To investigate whether HUWE1 regulates mitophagy in SH-SY5Y cells, we first assessed its subcellular localization following mitochondrial stress induced by oligomycin and antimycin A (O/A). Immunofluorescence analysis revealed a significant increase in HUWE1 co-localization with mitochondria upon O/A treatment (Fig. 1A). Consistently, biochemical fractionation confirmed elevated HUWE1 levels in the mitochondrial fraction after O/A exposure (Fig. 1B), indicating active recruitment of HUWE1 to damaged mitochondria. We then evaluated the functional contribution of HUWE1 to mitophagy. Overexpression of HUWE1 markedly enhanced mitophagosome formation, as evidenced by increased co-localization of LC3 with mitochondria following O/A treatment, compared with control cells (Fig. 1C). In parallel, HUWE1-overexpressing cells exhibited accelerated degradation of mitochondrial markers COX IV and TOMM20 relative to controls (Fig. 1D). Notably, overexpression of a catalytically inactive HUWE1 mutant (C4341S) failed to modulate mitophagy, demonstrating that the E3 ligase activity of HUWE1 is required for this effect. Conversely, HUWE1 knockdown via siRNA attenuated mitophagosome formation and impaired the O/A-induced degradation of COX IV and TOMM20 (Fig. 1E, F). Together, these findings demonstrate that HUWE1 is actively recruited to damaged mitochondria and promotes mitophagy in SH-SY5Y cells, with this function critically dependent on its E3 ubiquitin ligase activity.

### HUWE1 depletion sensitizes SH-SY5Y cells to 6-OHDA- and MPP⁺-induced cytotoxicity

**Fig. 2:**
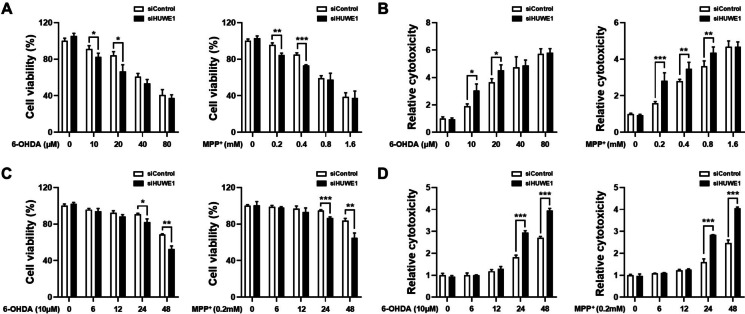
HUWE1 depletion increases SH-SY5Y cell susceptibility to 6-OHDA and MPP⁺. SH-SY5Y cells were transfected with control or *HUWE1* siRNA and treated with 6-OHDA or MPP⁺ at indicated concentrations and time points. (**A**) Cell viability measured by CCK-8 assay after 24 h exposure to increasing concentrations of 6-OHDA or MPP⁺. (**B**) Cytotoxicity measured by LDH release assay under increasing concentrations of 6-OHDA or MPP⁺. (**C**) Time-dependent cell viability measured by CCK-8 assay following treatment with 10 μM 6-OHDA or 0.2 mM MPP⁺. (**D**) Time-dependent cytotoxicity measured by LDH release assay following treatment with 10 μM 6-OHDA or 0.2 mM MPP⁺. Data are presented as mean ± SEM from at least three independent experiments. Statistical analysis was performed using one-way ANOVA followed by Tukey’s post hoc test. Statistical significance: *P < 0.05, **P < 0.01, ***P < 0.001

To assess whether HUWE1 depletion influences susceptibility to neurotoxin-induced cytotoxicity, SH-SY5Y cells were transfected with *HUWE1* siRNA and subsequently exposed to increasing concentrations of 6-OHDA or MPP⁺. At lower concentrations of 6-OHDA (10 and 20 μM), HUWE1-depleted cells exhibited a significant reduction in cell viability compared with control cells, whereas no substantial effect was observed at higher concentrations (40 and 80 μM). A similar pattern was observed with MPP⁺: HUWE1 knockdown decreased cell viability at lower doses (0.2 and 0.4 mM) but had minimal impact at higher doses (0.8 and 1.6 mM) (Fig. 2A). Consistent with these findings, HUWE1-depleted cells displayed enhanced cytotoxicity in response to both 6-OHDA and MPP⁺, as indicated by increased LDH release (Fig. 2B). Time-course analysis further revealed that HUWE1 depletion exacerbated the cytotoxic effects of low-dose 6-OHDA and MPP⁺ in a temporal manner relative to control cells (Fig. 2C, D). Collectively, these results indicate that HUWE1 depletion sensitizes SH-SY5Y cells to neurotoxin-induced cytotoxicity, particularly at lower concentrations of 6-OHDA and MPP⁺, underscoring the protective role of HUWE1 against dopaminergic neuronal stress.

### Neurotoxin exposure downregulates HUWE1 expression in SH-SY5Y cells

**Fig. 3:**
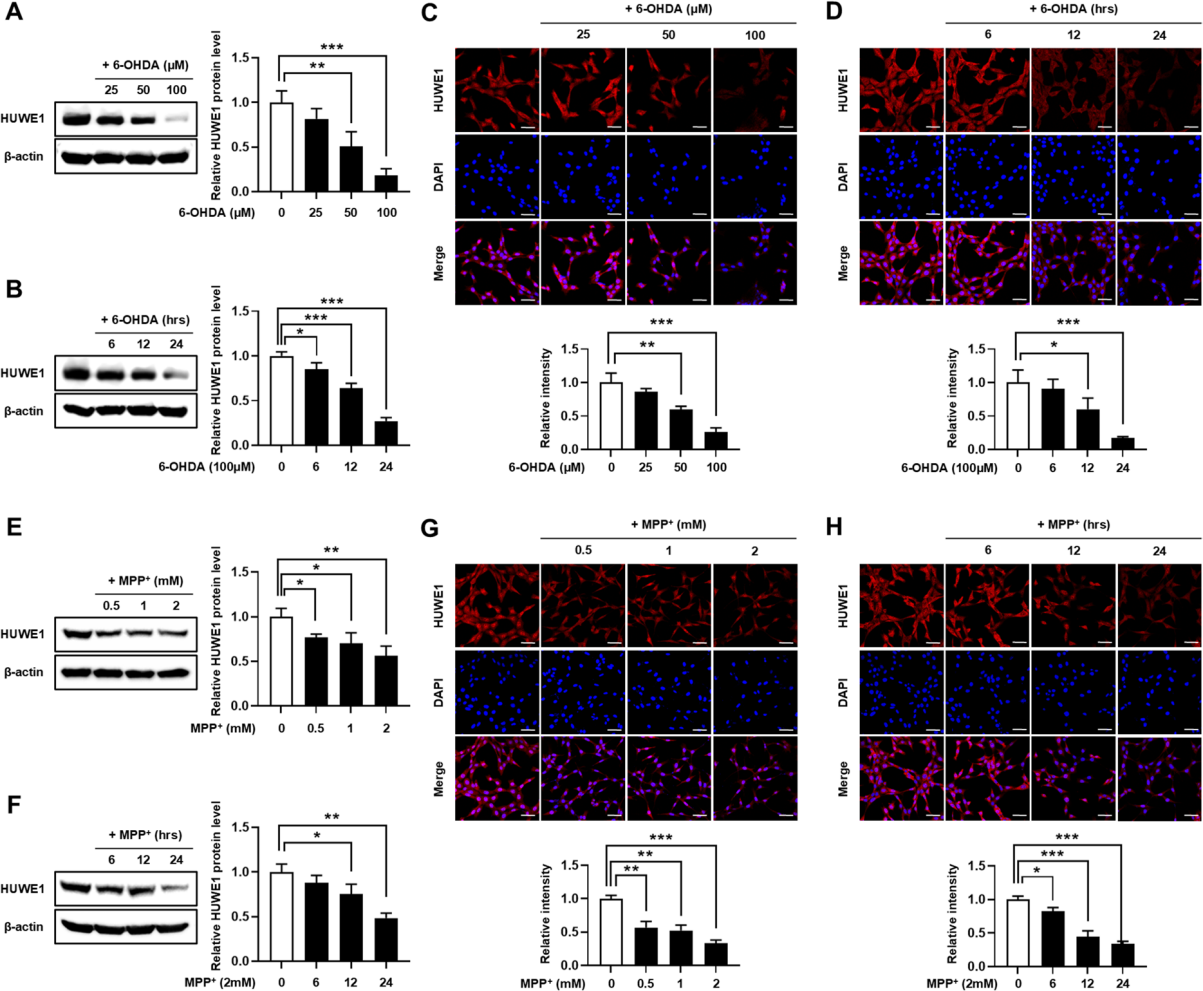
6-OHDA and MPP⁺ reduce HUWE1 expression in SH-SY5Y cells. SH-SY5Y cells were treated with 6-OHDA (25, 50, 100 μM) or MPP⁺ (0.5, 1, 2 mM) for 6, 12, or 24 h. (A, B) Western blot analysis of HUWE1 protein levels after 6-OHDA treatment at varying concentrations for 24 h (**A**) or at 100 μM for different time points (6, 12, 24 h) (**B**). (C, D) Immunofluorescence analysis of HUWE1 after 6-OHDA treatment at varying concentrations for 24 h (**C**) or at 100 μM for different time points (6, 12, 24 h) (**D**). Nuclei were stained with DAPI. Scale bar, 50 μm. (E, F) Western blot analysis of HUWE1 after MPP⁺ treatment at varying concentrations for 24 h (**E**) or at 2 mM for different time points (6, 12, 24 h) (**F**). (G, H) Immunofluorescence analysis of HUWE1 after MPP⁺ treatment at varying concentrations for 24 h (**G**) or at 2 mM for different time points (6, 12, 24 h) (**H**). Nuclei were stained with DAPI. Scale bar, 50 μm. Data are presented as mean ± SEM from at least three independent experiments. Statistical analysis was performed using one-way ANOVA followed by Tukey’s post hoc test. Statistical significance: *P < 0.05, **P < 0.01, ***P < 0.001

To determine whether neurotoxin exposure modulates HUWE1 expression, SH-SY5Y cells were treated with increasing concentrations of 6-OHDA or MPP⁺ for varying durations. Western blot analysis revealed a dose- and time-dependent reduction in HUWE1 protein levels upon 6-OHDA treatment (Fig. 3A, B). These observations were corroborated by immunofluorescence staining, which demonstrated decreased HUWE1 signal intensity in 6-OHDA-treated cells relative to controls (Fig. 3C, D). Similarly, MPP⁺ exposure led to a dose- and time-dependent decrease in HUWE1 expression, as confirmed by both Western blot and immunofluorescence analyses (Fig. 3E–H). Collectively, these results demonstrate that both 6-OHDA and MPP⁺ downregulate HUWE1 in SH-SY5Y cells in a concentration- and time-dependent manner.

### HUWE1 overexpression protects SH-SY5Y cells from 6-OHDA- and MPP⁺-induced cytotoxicity

**Fig. 4:**
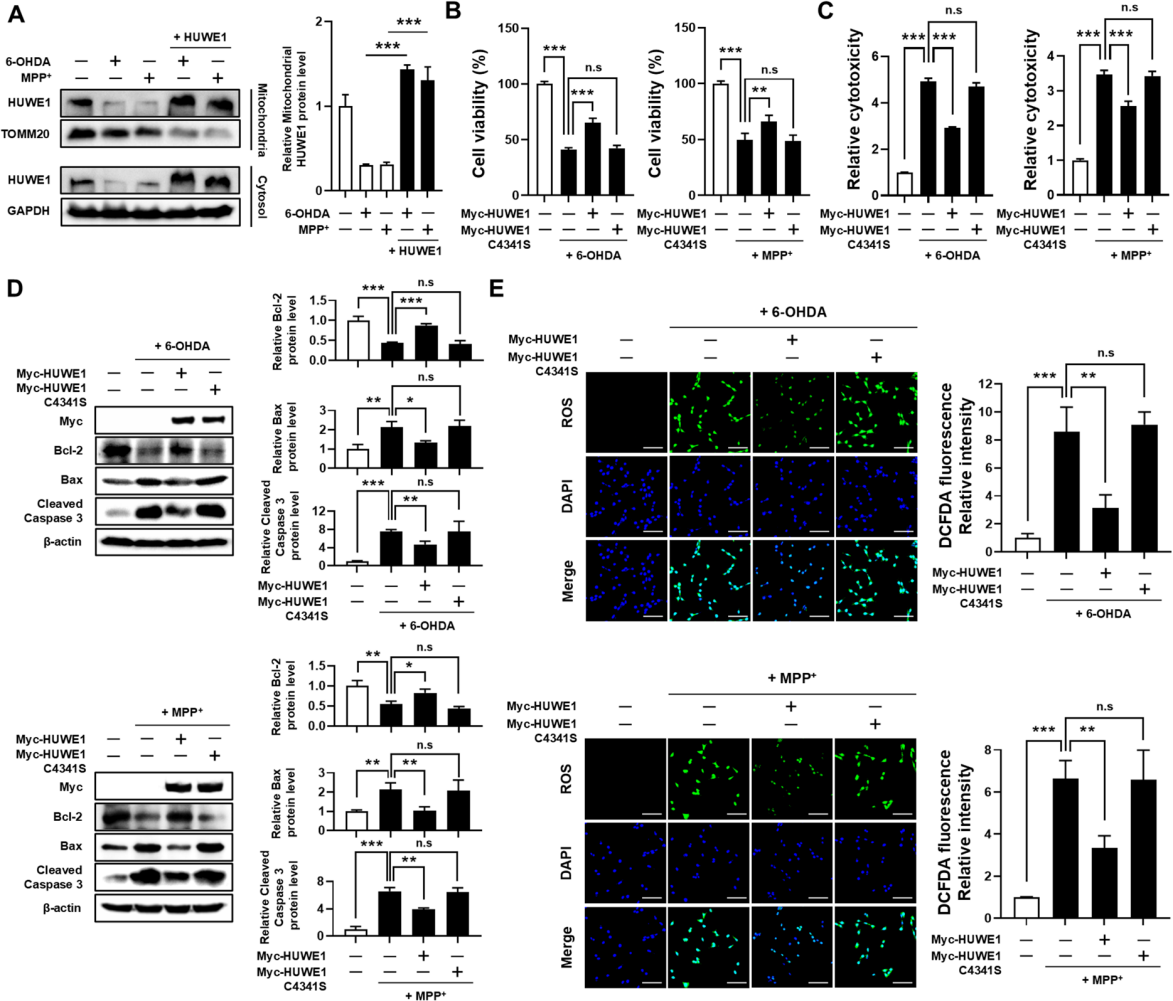
HUWE1 protects SH-SY5Y cells from 6-OHDA- and MPP⁺-induced cytotoxicity. SH-SY5Y cells were transfected with Myc-HUWE1 or catalytically inactive Myc-HUWE1^C4341S and then treated with 50 μM 6-OHDA or 1 mM MPP⁺ for 24 h. (**A**) Mitochondrial fractions were isolated and HUWE1 levels were analyzed by Western blot. (**B**) Cell viability measured by CCK-8 assay. (**C**) Cytotoxicity measured by LDH release assay. (**D**) Western blot analysis of apoptosis-related proteins (Bcl-2, Bax, Cleaved caspase-3). (**E**) Intracellular reactive oxygen species (ROS) levels detected by DCFDA staining. Scale bar, 100 μm. Data are presented as mean ± SEM from at least three independent experiments. Statistical analysis was performed using one-way ANOVA followed by Tukey’s post hoc test. Statistical significance: *P < 0.05, **P < 0.01, ***P < 0.001; n.s., not significant (*P* > 0.05)

To assess whether HUWE1 overexpression preserves its mitochondrial availability under neurotoxic stress, mitochondrial fractionation analyses were performed. Neurotoxin exposure (6-OHDA or MPP⁺) markedly reduced HUWE1 levels in the mitochondrial fraction, whereas HUWE1 overexpression restored mitochondrial HUWE1 abundance to near control levels (Fig. 4A). To evaluate the protective role of HUWE1 against neurotoxin-induced cytotoxicity, SH-SY5Y cells were transfected with plasmids encoding wild-type HUWE1. HUWE1 overexpression significantly rescued the reduction in cell viability caused by 6-OHDA and MPP⁺ compared to control cells (Fig. 4B). Consistently, LDH release assays indicated that HUWE1 overexpression attenuated 6-OHDA- and MPP⁺-induced cytotoxicity (Fig. 4C). Analysis of apoptosis-related proteins further corroborated the protective effect of HUWE1. Treatment with 6-OHDA or MPP⁺ decreased anti-apoptotic Bcl-2 levels and increased pro-apoptotic BAX and cleaved caspase-3 levels, indicative of apoptosis induction. HUWE1 overexpression restored these markers toward baseline levels, suggesting suppression of apoptosis (Fig. 4D). Additionally, HUWE1 overexpression mitigated neurotoxin-induced oxidative stress, as evidenced by reduced intracellular ROS accumulation following 6-OHDA or MPP⁺ treatment (Fig. 4E). In contrast, overexpression of a catalytically inactive HUWE1 C4341S mutant failed to protect cells from neurotoxin-induced cytotoxicity and oxidative stress, highlighting the requirement of HUWE1’s E3 ligase activity for its neuroprotective function.

### HUWE1 mitigates 6-OHDA- and MPP⁺-induced mitochondrial dysfunction in SH-SY5Y cells

**Fig. 5:**
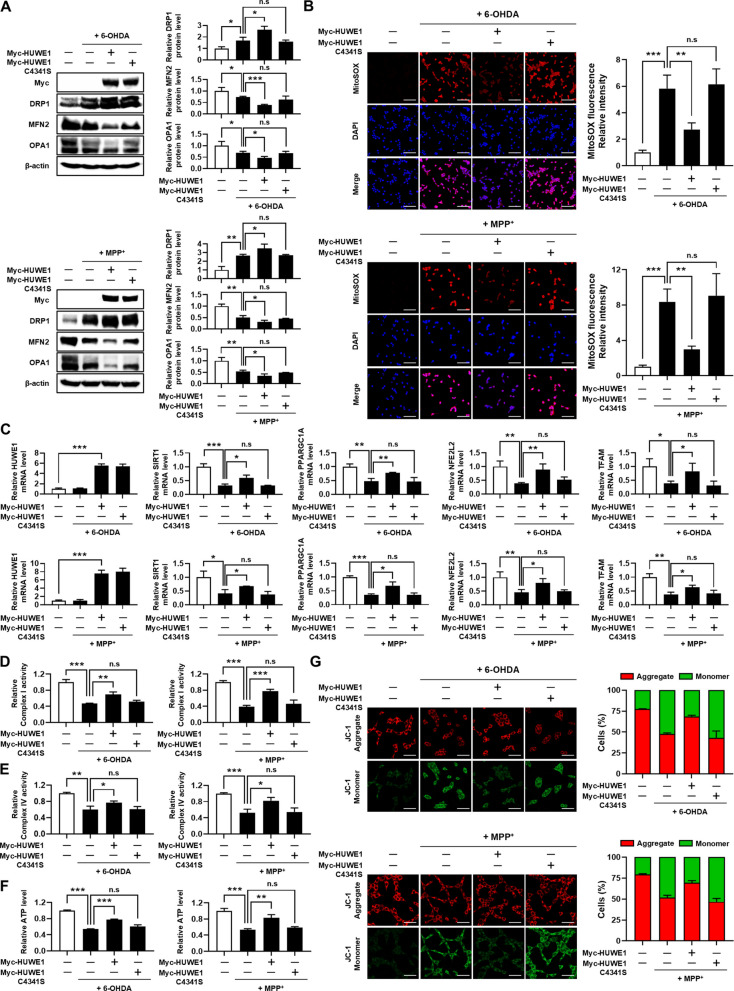
HUWE1 protects SH-SY5Y cells against 6-OHDA- and MPP⁺-induced mitochondrial dysfunction. SH-SY5Y cells were transfected with Myc-HUWE1 or catalytically inactive Myc-HUWE1^C4341S and treated with 50 μM 6-OHDA or 1 mM MPP⁺ for 24 h. (**A**) Western blot analysis of mitochondrial dynamics proteins DRP1, MFN2, and OPA1. (**B**) Mitochondrial reactive oxygen species (ROS) levels measured by MitoSOX Red staining. Scale bar, 100 μm. (**C**) qRT-PCR analysis of mRNA expression for *HUWE1*, *SIRT1*, *PPARGC1A*, *NFE2L2*, *and TFAM*. (**D**, **E**) Measurement of mitochondrial complex I and IV activities. (**F**) Measurement of intracellular ATP levels. (**G**) Mitochondrial membrane potential assessed by JC-1 staining. Scale bar, 50 μm. Data are presented as mean ± SEM from at least three independent experiments. Statistical analysis was performed using one-way ANOVA followed by Tukey’s post hoc test. Statistical significance: *P < 0.05, **P < 0.01, ***P < 0.001; n.s., not significant (*P* > 0.05)

Mitochondrial quality control during mitophagy relies on the dynamic regulation of mitochondrial fission and fusion. Fission segregates damaged mitochondria for selective autophagic removal, whereas fusion is suppressed to prevent incorporation of dysfunctional mitochondria into the healthy network (Twig et al. 2008; Youle & van der Bliek 2012). To examine whether HUWE1 influences mitochondrial dynamics under neurotoxic conditions, SH-SY5Y cells were transfected with HUWE1 or the catalytically inactive HUWE1 C4341S mutant and treated with 6-OHDA or MPP⁺. Under sustained neurotoxic conditions, mitochondrial dynamics exhibited a shift toward a fission-biased state, characterized by a modest increase in the fission-associated protein DRP1 and concomitant decreases in the fusion-related proteins MFN2 and OPA1. These alterations were limited in magnitude and are therefore interpreted as reflecting prolonged mitochondrial stress rather than direct regulation of mitochondrial dynamics proteins by neurotoxin exposure. HUWE1 overexpression further shifted this profile toward a fission-associated state, whereas the catalytically inactive HUWE1 C4341S mutant had no significant effect (Fig. 5A).

We further investigated the impact of HUWE1 on mitochondrial function. Overexpression of HUWE1 attenuated neurotoxin-induced mitochondrial ROS accumulation, indicating mitigation of oxidative stress (Fig. 5B). Additionally, HUWE1 restored the mRNA expression of mitochondrial biogenesis markers (*SIRT1, PPARGC1A, NFE2L2, TFAM*) that were downregulated by 6-OHDA and MPP⁺ (Fig. 5C). Functional analyses revealed that HUWE1 overexpression improved electron transport chain complex I and IV activities and ATP production that were impaired by neurotoxin exposure (Fig. 5D–F).

Given that mitochondrial membrane potential (MMP) is a key indicator of mitochondrial integrity (Agnihotri and Aruoma 2020; Kung et al. 2021), we assessed MMP using JC-1 staining. Low MMP results in green fluorescence, while high MMP generates red fluorescence (Cai et al. 2008). 6-OHDA and MPP⁺ decreased the red/green fluorescence ratio, indicative of MMP loss, whereas HUWE1 overexpression restored this ratio compared with control cells. In contrast, the HUWE1 C4341S mutant failed to rescue MMP (Fig. 5G). Collectively, these findings demonstrate that HUWE1 mitigates 6-OHDA- and MPP⁺-induced mitochondrial dysfunction in SH-SY5Y cells by promoting mitophagy-dependent mitochondrial quality control, thereby preserving mitochondrial integrity, electron transport chain activity, and ATP production.

### BL-918 activates HUWE1-mediated mitophagy via AMBRA1 recruitment in SH-SY5Y cells

**Fig. 6:**
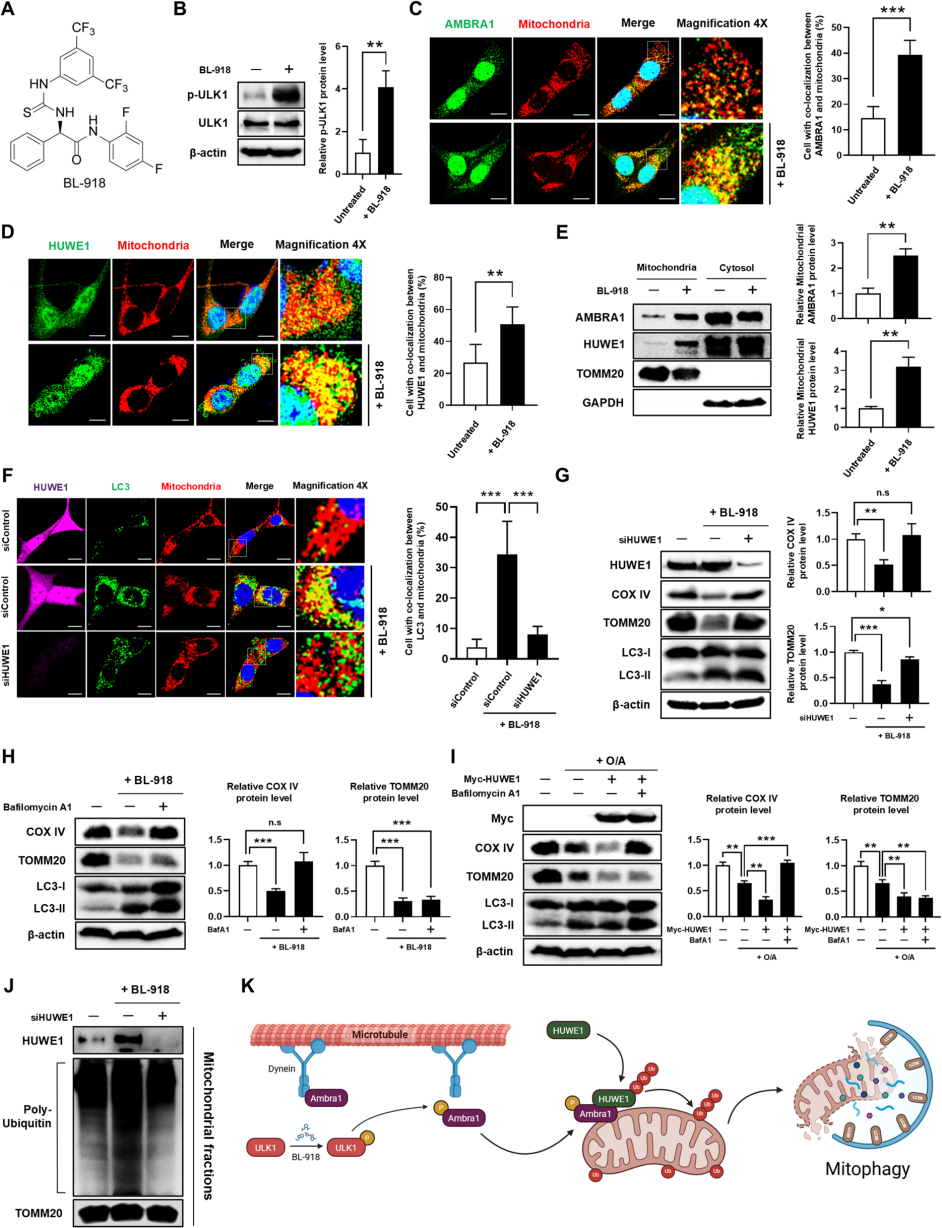
BL-918 activates HUWE1-mediated mitophagy. (**A**) Chemical structure of BL-918. (B–E) SH-SY5Y cells were treated with 20 μM BL-918. (**B**) Western blot analysis of phospho-ULK1 and ULK1. (C, D) Immunofluorescence analysis of AMBRA1 (**C**) or HUWE1 (**D**) co-localization with mitochondria after 8 h. Scale bar, 10 µm. (**E**) Western blot analysis of AMBRA1 and HUWE1 in mitochondrial fractions after 24 h. (F–G) SH-SY5Y cells transfected with *HUWE1* siRNA were treated with 20 μM BL-918. (**F**) Immunofluorescence analysis of LC3–mitochondria co-localization after 8 h. Scale bar, 10 µm. (**G**) Western blot analysis of HUWE1, COX IV, TOMM20, and LC3. (**H**) SH-SY5Y cells were treated with BL-918 in the presence or absence of bafilomycin A1 for 24 h, and COX IV and TOMM20 levels were analyzed by western blot. (**I**) SH-SY5Y cells overexpressing HUWE1 were treated with O/A for 8 h in the presence or absence of bafilomycin A1, and COX IV and TOMM20 levels were analyzed western blot. (**J**) SH-SY5Y cells transfected with *HUWE1* siRNA were treated with 20 μM BL-918. Western blot analysis of mitochondrial ubiquitination; cells were pretreated with MG132 for 6 h. (**K**) Schematic model of BL-918–induced HUWE1-mediated mitophagy (created with BioRender). Data are presented as mean ± SEM from at least three independent experiments. Panels B–E were analyzed using an unpaired two-tailed Student’s t-test, whereas panels F–J were analyzed using one-way ANOVA followed by Tukey’s post hoc test. Statistical significance: *P < 0.05, **P < 0.01, ***P < 0.001; n.s., not significant (*P* > 0.05)

To explore potential modulators of HUWE1-mediated mitophagy, we investigated BL-918, a pharmacological activator of ULK1. Although direct HUWE1 activators have not been reported, ULK1 activation by BL-918 can promote HUWE1 function indirectly through phosphorylation of AMBRA1, facilitating its translocation to mitochondria-associated membranes (MAMs) and subsequent induction of mitophagy (Li et al. 2022; Liu et al. 2023) (Fig. 6A). We first assessed whether BL-918 treatment recruits AMBRA1 and HUWE1 to mitochondria in SH-SY5Y cells. BL-918 enhanced ULK1 phosphorylation (Fig. 6B), and immunofluorescence analysis revealed increased co-localization of AMBRA1 and HUWE1 with mitochondria (Fig. 6C, D). Consistently, mitochondrial fractionation analyses revealed that BL-918 treatment markedly enhanced the recruitment of both AMBRA1 and HUWE1 to the mitochondrial fraction (Fig. 6E). To determine whether BL-918 activates mitophagy in a HUWE1-dependent manner, mitophagic activity was compared between control and HUWE1-depleted cells. In control cells, BL-918 significantly increased mitophagosome formation, as indicated by enhanced co-localization of LC3 with mitochondria, whereas this effect was markedly attenuated in HUWE1-depleted cells (Fig. 6F). Similarly, BL-918 induced degradation of mitochondrial markers COX IV and TOMM20 in control cells, which was substantially reduced upon HUWE1 knockdown (Fig. 6G). To assess whether the reduction of mitochondrial proteins was mediated by autophagic–lysosomal degradation, cells were treated with the lysosomal inhibitor bafilomycin A1 (Yoshimori et al. 1991; Mauvezin and Neufeld 2015). Bafilomycin A1 treatment prevented the BL-918–induced reduction of the inner mitochondrial membrane protein COX IV and led to accumulation of LC3-II, indicating inhibition of autophagic flux (Fig. 6H). In contrast, the reduction of the outer mitochondrial membrane protein TOMM20 was not significantly affected by bafilomycin A1 treatment. Similarly, HUWE1 overexpression under O/A treatment reduced COX IV protein levels, whereas this reduction was abolished in the presence of bafilomycin A1 (Fig. 6I).

To further examine whether HUWE1 is involved in the ubiquitination of mitochondrial proteins prior to their degradation, mitochondrial ubiquitination was assessed under proteasome inhibition using MG132. BL-918 treatment increased ubiquitination of mitochondrial proteins in control cells; however, this increase was no longer observed in HUWE1-depleted cells (Fig. 6J). Collectively, these results demonstrate that BL-918 activates HUWE1-mediated mitophagy in SH-SY5Y cells by promoting ULK1-dependent phosphorylation, facilitating AMBRA1 recruitment to mitochondria, and enhancing HUWE1-dependent ubiquitination of outer mitochondrial membrane proteins, culminating in lysosome-dependent mitochondrial turnover (Fig. 6K). These findings suggest that BL-918 functions as an indirect activator of HUWE1-mediated mitophagy and may serve as a potential neuroprotective agent under conditions of mitochondrial stress.

### BL-918 attenuates 6-OHDA- and MPP⁺-induced neurotoxicity via HUWE1 activation in SH-SY5Y cells

**Fig. 7:**
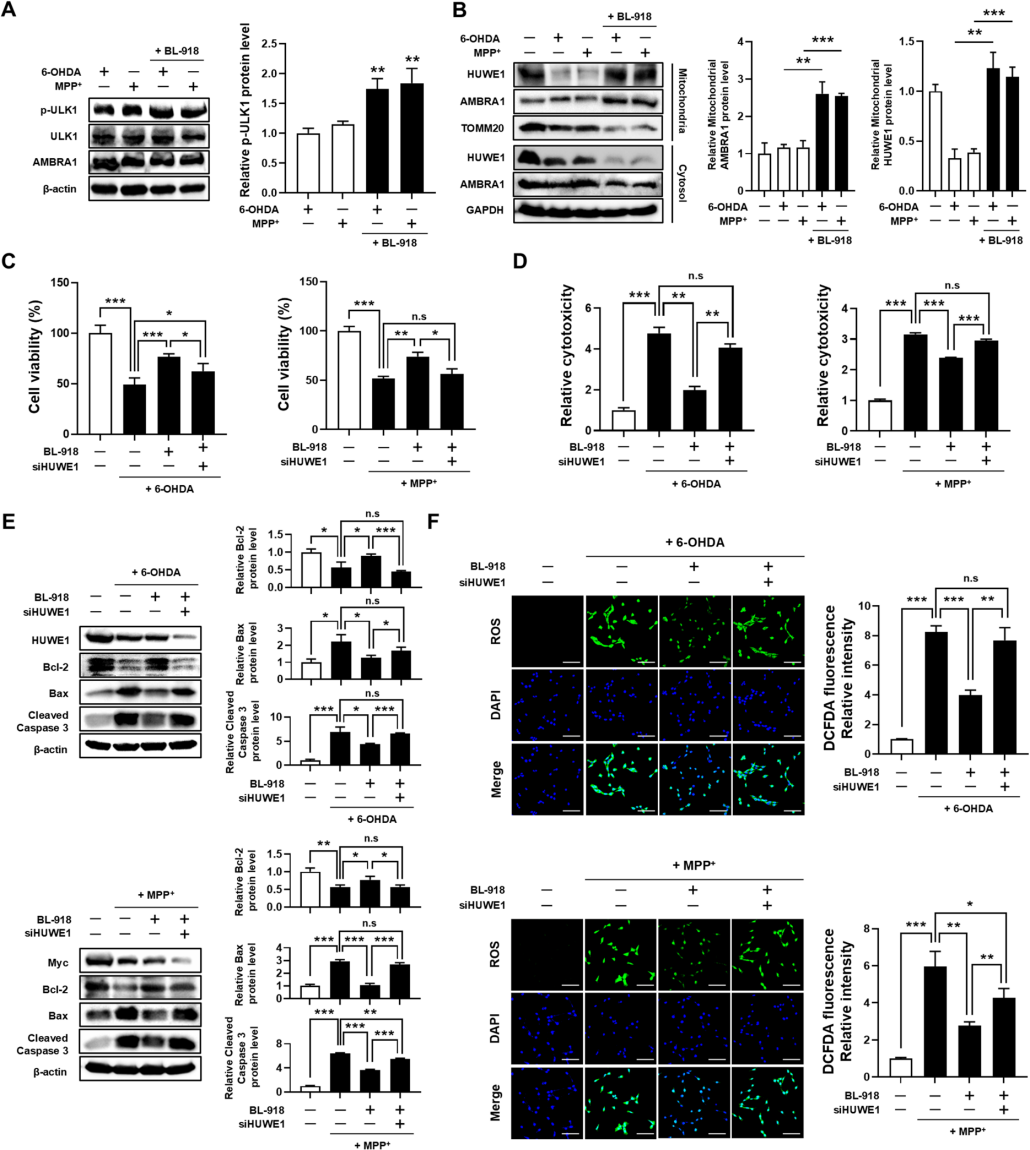
BL-918 protects SH-SY5Y cells against 6-OHDA- and MPP⁺-induced cytotoxicity in a HUWE1-dependent manner. SH-SY5Y cells, transfected with negative control or *HUWE1* siRNA, were treated with 50 μM 6-OHDA or 1 mM MPP⁺ for 24 h in the presence or absence of 20 μM BL-918. (**A**) Western blot analysis of phospho-ULK1, total ULK1, and AMBRA1. (**B**) Western blot analysis of AMBRA1 and HUWE1 in mitochondrial fractions. (**C**) Cell viability measured by CCK-8 assay. (**D**) Cytotoxicity measured by LDH release assay. (**E**) Western blot analysis of apoptosis-related proteins (Bcl-2, Bax, Cleaved caspase-3). (**F**) Intracellular ROS levels measured by DCFDA staining. Scale bar, 100 μm. Data are presented as mean ± SEM from at least three independent experiments. Statistical analysis was performed using one-way ANOVA followed by Tukey’s post hoc test. Statistical significance: *P < 0.05, **P < 0.01, ***P < 0.001; n.s., not significant (*P* > 0.05)

To investigate whether BL-918-mediated activation of HUWE1 confers neuroprotection under neurotoxic conditions, SH-SY5Y cells were treated with 6-OHDA or MPP⁺ in the presence or absence of BL-918. BL-918 treatment significantly increased ULK1 phosphorylation and enhanced mitochondrial recruitment of HUWE1 in neurotoxin-treated cells (Fig. 7A, B), indicating that BL-918 effectively engages the HUWE1-mediated mitophagy pathway under stress conditions. We next assessed whether BL-918 confers cytoprotection via HUWE1-mediated mitophagy. BL-918 treatment significantly improved cell viability, which was otherwise reduced by 6-OHDA or MPP⁺ (Fig. 7C), and attenuated cytotoxicity compared with neurotoxin treatment alone (Fig. 7D). Consistently, BL-918 restored apoptosis-related protein expression: it reversed the neurotoxin-induced decrease in Bcl-2 and the increase in BAX and cleaved caspase-3 levels (Fig. 7E). Furthermore, BL-918 markedly suppressed intracellular ROS accumulation induced by 6-OHDA and MPP⁺ (Fig. 7F). Notably, the protective effects of BL-918 were abolished in HUWE1-depleted cells, demonstrating that BL-918 confers cytoprotection in a HUWE1-dependent manner. These findings collectively indicate that BL-918 mitigates neurotoxin-induced oxidative stress and apoptotic cell death by activating HUWE1-mediated mitophagy.

### BL-918 protects mitochondrial integrity under 6-OHDA and MPP⁺ stress via HUWE1

**Fig. 8:**
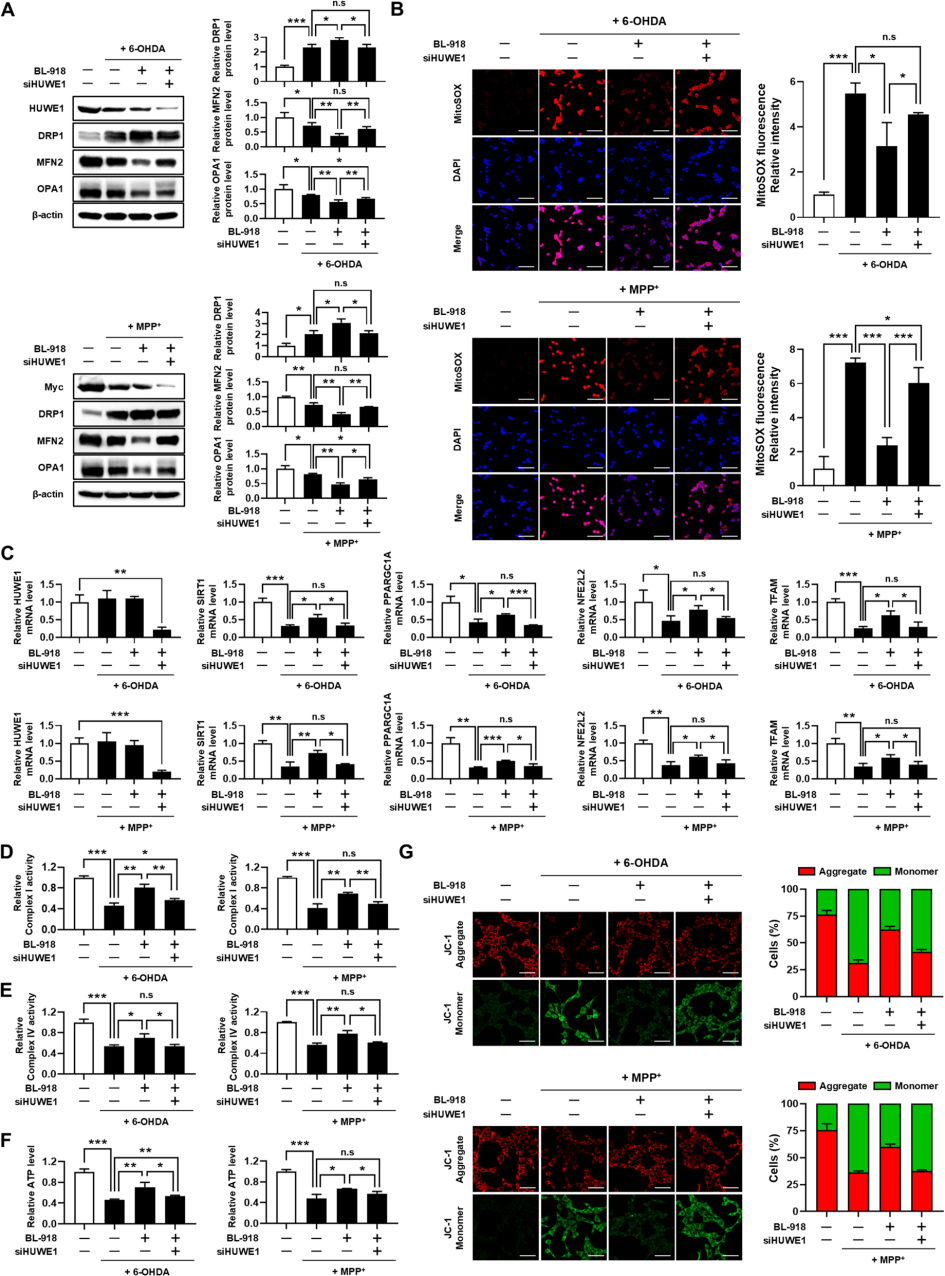
BL-918 protects SH-SY5Y cells against 6-OHDA- and MPP⁺-induced mitochondrial dysfunction in a HUWE1-dependent manner. SH-SY5Y cells, transfected with control or *HUWE1* siRNA, were treated with 50 μM 6-OHDA or 1 mM MPP⁺ for 24 h in the presence of 20 μM BL-918. (**A**) Western blot analysis of mitochondrial dynamics proteins DRP1, MFN2, and OPA1. (**B**) Mitochondrial ROS levels measured by MitoSOX Red staining. Scale bar, 100 μm. (**C**) qRT-PCR analysis of *HUWE1*, *SIRT1*, *PPARGC1A*, *NFE2L2*, and *TFAM* mRNA expression. (**D**, **E**) Activities of mitochondrial complex I and IV. (**F**) Intracellular ATP levels. (**G**) Mitochondrial membrane potential assessed by JC-1 staining. Scale bar, 50 μm. Data are presented as mean ± SEM from at least three independent experiments. Statistical analysis was performed using one-way ANOVA followed by Tukey’s post hoc test. Statistical significance: *P < 0.05, **P < 0.01, ***P < 0.001; n.s., not significant (*P* > 0.05)

To evaluate whether BL-918 mitigates mitochondrial dysfunction induced by 6-OHDA and MPP⁺ through HUWE1-mediated mitophagy, SH-SY5Y cells were treated with neurotoxins in the presence or absence of BL-918. BL-918 treatment promoted the expression of the mitochondrial fission protein DRP1 while suppressing fusion proteins MFN2 and OPA1 under neurotoxic conditions, indicating enhanced mitochondrial fission and inhibited fusion. This shift suggests that BL-918 facilitates the segregation of damaged mitochondria, thereby promoting their selective clearance via mitophagy (Fig. 8A). Furthermore, BL-918 significantly reduced mitochondrial ROS accumulation induced by 6-OHDA and MPP⁺ (Fig. 8B) and restored the mRNA expression of mitochondrial biogenesis markers that were downregulated by neurotoxin exposure (Fig. 8C). Consistently, BL-918 improved neurotoxin-induced impairments in mitochondrial ETC activity and ATP production (Fig. 8D-F). Assessment of MMP using JC-1 staining demonstrated that BL-918 increased the red/green fluorescence ratio, indicating preserved MMP under 6-OHDA and MPP⁺ stress (Fig. 8G). Importantly, the protective effects of BL-918 on mitochondrial dynamics, oxidative stress, biogenesis, ETC activity, ATP production, and MMP were all abrogated upon HUWE1 depletion. These results demonstrate that BL-918 preserves mitochondrial quality and function under neurotoxic stress through a HUWE1-dependent mitophagy mechanism.

## Discussion

Mitochondrial homeostasis is essential for neuronal survival, as neurons are highly energy-demanding and particularly vulnerable to oxidative stress and mitochondrial dysfunction. Mitophagy, the selective autophagic clearance of damaged mitochondria, represents a critical quality control mechanism in this context. Historically, research on neuronal mitophagy has focused on the PINK1/Parkin pathway, the canonical regulator of mitochondrial clearance (Narendra et al. 2010; Matsuda et al. 2010; Vincow et al. 2013). Activation of Parkin has been shown to confer neuroprotection in various models of PD, including neurotoxin-induced models (Chung et al. 2020; Wang et al. 2021; Lu et al. 2023). However, the majority of PD cases are sporadic, lacking PINK1 or Parkin mutations, and therapeutic interventions targeting this pathway alone have shown limited efficacy. This limitation has led to growing interest in PINK1/Parkin-independent mitophagy mechanisms, such as SIRT1/AMPK/mTOR and BNIP3/NIX-mediated pathways, which have been demonstrated to alleviate mitochondrial stress in neuronal contexts (Chen et al. 2021, 2022). These findings underscore the need to identify novel mitophagy regulators that operate independently of canonical pathways, particularly in sporadic PD.

In this study, we identified HUWE1, a HECT-type E3 ubiquitin ligase, as a previously unrecognized regulator of mitophagy in neuronal cells. We demonstrated that HUWE1 is recruited to the outer mitochondrial membrane upon mitochondrial damage, thereby promoting mitophagic clearance and ultimately conferring neuroprotection. The neuroprotective relevance of HUWE1 has so far been suggested only by a small number of clinical observations reporting loss-of-function mutations associated with neurological disorders such as intellectual disability, epilepsy, and neurodevelopmental delay (Moortgat et al. 2018; van Eyk et al. 2018; Takata et al. 2019; Johannesen et al. 2015). Our findings therefore provide the first direct experimental evidence that HUWE1 regulates both neuronal survival and mitophagy, representing a significant conceptual advance beyond prior clinical correlations.

Functionally, HUWE1 protected SH-SY5Y cells from 6-OHDA- and MPP⁺-induced mitochondrial dysfunction, oxidative stress, and apoptosis. Interestingly, exposure to neurotoxins led to a marked downregulation of HUWE1 expression, suggesting that toxic stress not only induces direct mitochondrial injury but also suppresses intrinsic protective mechanisms. This raises the possibility of a feed-forward loop, in which mitochondrial dysfunction further reduces HUWE1 expression, thereby impairing mitophagy and exacerbating neuronal vulnerability. Supporting this model, overexpression of functional HUWE1, but not its catalytically inactive mutant, protected against both toxin-induced mitochondrial damage and neuronal loss. However, it remains unclear whether HUWE1 downregulation represents a direct consequence of neurotoxin exposure, a secondary effect of mitochondrial injury, or whether HUWE1 independently regulates both mitochondrial integrity and neuronal survival. Future studies in which mitochondrial injury is pharmacologically or genetically suppressed will be essential to determine whether neurotoxins directly modulate HUWE1 expression or whether these events are mechanistically connected in a self-reinforcing cycle.

Importantly, although HUWE1 overexpression restored mitochondrial respiratory capacity and ATP production under neurotoxic conditions, these effects should not be interpreted as direct reversal or repair of neurotoxin-induced mitochondrial damage. Rather, our data support a model in which HUWE1 overexpression expands the total HUWE1 pool and restores its functional availability at mitochondria, thereby enhancing HUWE1-dependent mitophagy. Through the selective clearance of severely damaged mitochondria, the overall quality of the mitochondrial population is improved, which secondarily preserves mitochondrial function at the cellular level. This interpretation is further supported by lysosomal inhibition experiments demonstrating intact autophagic flux, indicating productive mitophagy rather than impaired degradation.

Our findings expand the functional scope of HUWE1 beyond its previously reported roles in cancer and proliferative cells, where it has been implicated in mitophagy and metabolic regulation (Kao et al. 2018; Gong et al. 2020; Qi et al. 2022). Unlike proliferating cells, post-mitotic neurons rely heavily on robust mitochondrial quality control to sustain bioenergetic homeostasis over a lifetime. The identification of HUWE1 as a regulator of mitophagy in this context highlights the physiological relevance of non-canonical mitophagy pathways and provides new mechanistic insight into neuronal mitochondrial quality control under stress conditions.

Our results demonstrate that HUWE1 functions downstream of AMBRA1 recruitment, establishing a mechanistic link between ULK1-AMBRA1 signaling and E3 ligase-mediated ubiquitination in mitophagy. While previous studies have implicated AMBRA1-mediated mitophagy in protecting neurons against oxidative stress and apoptosis (Di Rita et al. 2018b), the downstream regulation of this pathway remained largely unclear. Here, we provide evidence that HUWE1 mediates AMBRA1-dependent mitophagy, highlighting a central axis in the regulation of mitochondrial quality control in neurons. Although direct pharmacological activators of HUWE1 are currently unavailable, we offer proof-of-concept that targeting upstream regulators can enhance HUWE1 activity. Treatment with BL-918, an ULK1 activator, promoted AMBRA1 phosphorylation and mitochondrial translocation, facilitating HUWE1-mediated ubiquitination of mitochondrial substrates. BL-918 restored mitochondrial dynamics, reduced reactive oxygen species accumulation, preserved ATP production, and maintained ETC activity under neurotoxic conditions. Notably, these protective effects were abolished upon HUWE1 depletion, confirming the specificity of this mechanism. Together, these findings suggest that the ULK1–AMBRA1–HUWE1 axis represents a functionally relevant mechanism for regulating mitophagy in neuronal cells under neurotoxic stress.

However, this study has several limitations. First, all experiments were conducted in SH-SY5Y cells, which, although widely used as a dopaminergic neuronal model (Xicoy et al. 2017), do not fully capture the complexity of in vivo neuronal circuits or the heterogeneous cellular environment of the brain. To confirm the physiological relevance of HUWE1 in neuronal survival and mitophagy, further studies using primary neuronal cultures and in vivo models are required. Second, although multiple complementary lines of evidence support HUWE1-mediated mitophagy—including increased LC3–mitochondrial colocalization, accelerated turnover of mitochondrial proteins, and lysosomal inhibition experiments demonstrating lysosome-dependent degradation of COX IV (with early proteasome involvement in TOMM20 processing)—the incorporation of dedicated mitophagy reporters, such as mito-Keima, would enable more quantitative assessment of mitochondrial delivery to acidic lysosomes and further refine measurements of mitophagic flux. Additionally, our assessment of mitochondrial morphology was based on Airyscan-enhanced confocal imaging, which is sufficient to evaluate stress-associated mitochondrial integrity and dysfunction but does not resolve fine ultrastructural features of the mitochondrial network achievable by super-resolution techniques such as STED or SIM; therefore, our conclusions regarding mitochondrial dynamics were intentionally limited to fission- or fusion-biased shifts supported by imaging and biochemical data. Third, while we demonstrate that HUWE1 overexpression protects against neurotoxin-induced mitochondrial dysfunction and neuronal damage, the therapeutic potential of enhancing HUWE1 function has not yet been evaluated in animal models of PD. At present, no specific HUWE1 activators are available; therefore, the generation of transgenic animal models will be essential to further investigate the in vivo role of HUWE1 and its therapeutic potential. Notably, the exceptionally large size of the HUWE1 gene poses challenges for efficient delivery in animal models or potential clinical applications (Boye et al. 2022). Strategies to overcome this limitation may include the development of small molecules that enhance HUWE1-mediated ubiquitination or the design of functional truncated variants lacking non-essential domains. Fourth, the precise mechanisms regulating HUWE1 remain incompletely understood. Although we show that neurotoxin exposure downregulates HUWE1 expression, it is unclear whether this effect is direct or mediated via mitochondrial injury. In addition, our study does not clarify how HUWE1 interacts with other mitophagy regulators such as PINK1 and Parkin, or whether it functions in parallel, non-canonical pathways of mitochondrial quality control. Systematic characterization of HUWE1 regulation in sporadic PD patient-derived neurons and postmortem brain tissue could provide critical insights into its role in disease pathogenesis.

In conclusion, our study identifies HUWE1 as a critical regulator of mitophagy and neuronal survival, providing the first direct evidence of its neuroprotective role in the context of PD-related neurotoxicity. By facilitating the ubiquitination of damaged mitochondrial proteins, HUWE1 promotes mitochondrial quality control, and its loss or downregulation may exacerbate neuronal vulnerability through a potential feed-forward mechanism. These findings expand the functional scope of HUWE1 beyond its established roles in proliferative cells, highlighting the importance of non-canonical mitophagy pathways in neurons. While further studies in primary neurons and in vivo models are necessary to validate its therapeutic potential, HUWE1 emerges as a promising target for strategies aimed at preserving mitochondrial integrity and mitigating neurodegeneration.

## Data Availability

No datasets were generated or analysed during the current study.
